# Preventive health care in blood cancer survivors: results from the ABC study

**DOI:** 10.1007/s00432-023-04984-9

**Published:** 2023-07-03

**Authors:** Julia Baum, Hildegard Lax, Nils Lehmann, Anja Merkel-Jens, Dietrich W. Beelen, Karl-Heinz Jöckel, Ulrich Dührsen

**Affiliations:** 1 Klinik für Hämatologie, Universitätsklinikum Essen, Universität Duisburg-Essen, Hufelandstraße 55, 45147 Essen, Germany; 2 Institut für Medizinische Informatik, Biometrie und Epidemiologie, Universität Duisburg-Essen, Essen, Germany; 3 Klinik für Knochenmarktransplantation, Universitätsklinikum Essen, Universität Duisburg-Essen, Essen, Germany

**Keywords:** Allogeneic transplantation, Blood cancer, Cancer screening, Cardiovascular risk factors, Follow-up care, Preventive health care, Vaccination

## Abstract

**Background:**

Blood cancer survivors are at increased risk for second primary malignancies, cardiovascular diseases, and infections. Little is known about preventive care in blood cancer survivors.

**Methods:**

Our questionnaire-based study included blood cancer patients diagnosed at the University Hospital of Essen before 2010, with a ≥ 3-year interval from the last intense treatment. One section of the retrospective study covered preventive care (cancer screening, cardiovascular screening, vaccination).

**Results:**

Preventive care was delivered by a general practitioner for 1100 of 1504 responding survivors (73.1%), by an oncologist for 125 (8.3%), by a general practitioner together with an oncologist for 156 (10.4%), and by other disciplines for 123 (8.2%). Cancer screening was more consistently performed by general practitioners than by oncologists. The converse was true for vaccination, with particularly high vaccination rates in allogeneic transplant recipients. Cardiovascular screening did not differ between care providers. Cancer and cardiovascular screening rates in survivors eligible for statutory prevention programs were higher than in the general population (skin cancer screening 71.1%; fecal occult blood testing 70.4%; colonoscopy 64.6%; clinical breast examination 92.1%; mammography 86.8%; cervical smear 86.0%; digital rectal examination 61.9%; blood pressure test 69.4%; urine glucose test 54.4%; blood lipid test 76.7%; information about overweight 71.0%). The *Streptococcus pneumoniae* vaccination rate was higher (37.0%) and the influenza vaccination rate was lower (57.0%) than in the general population.

**Conclusions:**

Utilization of preventive care is high among German blood cancer survivors. To ensure widespread delivery and avoid redundancy, communication between oncologists and preventive care providers is essential.

**Supplementary Information:**

The online version contains supplementary material available at 10.1007/s00432-023-04984-9.

## Background

Due to improvements in diagnosis and treatment, the number of long-term cancer survivors is continuously growing (Allemani et al. 2018; Lagergren et al. 2019). Because of increased risks for adverse physical, mental and social consequences, follow-up care is an important component of long-term support (Jacobs and Shulman 2017). How best to provide this support is a matter of debate (Jefford et al. 2022).

For blood cancer, the fourth most common cancer in men and women (Smith et al. 2011; Parry et al. 2011), little is known about follow-up care (Laidsaar-Powell et al. 2019). Follow-up care focusses on a single disease diagnosed in the past and currently in remission, not requiring therapy, or controlled by measures not significantly interfering with everyday activities. It is based on scheduled consultations with a follow-up physician. To what extent these consultations interfere with other aspects of health care, is largely unknown (Ng et al. 2008; Hodgson et al. 2010). While long-term cancer- or cancer treatment-related sequelae and other acute or chronic illnesses are diagnosed and treated by specialists from the respective fields (Emery et al. 2022), the patients’ attitude towards preventive health care and the physicians providing it may change after a blood cancer diagnosis.

To gather information about follow-up care received by patients diagnosed and treated at the University Hospital of Essen, the oldest and one of the largest comprehensive cancer centers in Germany, we performed the ‘Aftercare in Blood Cancer Survivors’ (ABC) study (Baum et al. 2023). One section of the study’s retrospective part focused on utilization of preventive health care by blood cancer survivors.

## Methods

### Eligibility

Patients 18 years or older diagnosed with and/or treated for a hematological malignancy at the University Hospital of Essen were eligible for the study, provided that the interval between study inclusion and the date of diagnosis (for untreated patients) or the end of last treatment (for primary disease, relapse, or a second primary malignancy) was ≥ 3 years. In patients receiving continuous oral medication or low dose maintenance after intensive induction therapy, eligibility started 3 years after treatment initiation or end of induction, respectively. Patients exclusively treated in childhood or adolescence were not eligible.

### Study design

The ABC study was an observational study. It was performed from October 2013 to December 2016 and comprised a 6-month retrospective and an 18-month prospective part. The study was approved by the ethics committee of the University of Duisburg-Essen (no. 14-5692-BO).

In the retrospective part, eligible patients were identified by hospital documents, spanning the period from 1999 to 2010 (Baum et al. 2023). Patients were informed by mail about the purpose of the study and invited to complete a 118-item questionnaire specifically designed for the study. Nineteen questions pertained to general health care and prevention (see Online Supplementary Material). Patients not responding to our invitation within 4–6 weeks were contacted by mail again, and patients failing to respond to the second invitation were reminded by phone.

### Statistical analysis

Survivors were categorized according to treatment of the hematologic malignancy (no allogeneic transplantation versus transplantation) and the physician providing preventive care (general practitioner alone versus oncologist alone versus general practitioner and oncologist). Participation in statutory prevention programs was restricted to survivors beyond the age limit of the respective programs (≥ 20 years, cervical smear; ≥ 30 years, clinical breast examination; ≥ 35 years, skin cancer screening, cardiovascular screening; ≥ 45 years, digital rectal examination; ≥ 50 years, mammography, fecal occult blood test, colonoscopy (males); ≥ 55 years, colonoscopy (females); ≥ 60 years, vaccination against influenza and *Streptococcus pneumoniae*). Patients were categorized according to the timepoint of becoming eligible for the statutory prevention program (before or after the blood cancer diagnosis).

The main outcome variable was the screening or vaccination rate. Frequencies are presented as numbers and compared using the chi^2^ test. Comparisons included survivors with or without a history of allogeneic transplantation, survivors treated by different preventive care providers, and survivors becoming eligible for a statutory prevention program before or after the blood cancer diagnosis. All analyses are exploratory, assuming statistical significance at p ≤ 0.05.

## Results

### Patients and follow-up institutions

Of 2555 patients contacted, 841 men and 710 women participated in the study. The median age was 58 years (range 23–91), and the median time from last treatment was 9 years (range 3–36). The patients were allocated to seven groups of diseases not treated by allogeneic transplantation and one allogeneic transplantation group comprising all 554 transplanted patients irrespective of the underlying disease (Table 1). Details have been described before (Baum et al. 2023).

**Table 1:** Patient characteristics (Baum et al. 2023)

Total number of patients	1551
No allogeneic transplantation, number of patients (% of total number)	997 (64.3%)
MGUS	19
MM	37
iNHL/CLL	264
MPN/CML	107
MDS	5
aNHL/HL	491
AML/ALL	74
Allogeneic transplantation^a^, number of patients (% of total number)	554 (35.7%)
Age at study entry—years, median (range)	57.6 (23.0–91.2)
Time from diagnosis—years, median (range)	10.5 (3.0–40.7)
Time from last treatment^b^—years, median (range)	8.9 (3.0–36.0)
Male, number of patients (% of total number)	841 (54.2%)
Female, number of patients (% of total number)	710 (45.8%)
Follow-up period, number of patients (% of total number)	
Year 4–5	292 (18.8%)
Year 6–10	528 (34.1%)
Year > 10	731 (47.1%)
Follow-up institutions, number of patients (% of total number)	
University Hospital of Essen	1045 (67.4%)
External oncologist	231 (14.9%)
Internist/practitioner^c^	203 (13.1%)
No follow-up	72 (4.6%)

*MGUS* monoclonal gammopathy of undetermined significance, *MM* multiple myeloma, *iNHL* indolent non-Hodgkin lymphoma, *CLL* chronic lymphocytic leukemia, *MPN* myeloproliferative neoplasm, *CML* chronic myeloid leukemia, *MDS* myelodysplastic syndrome, *aNHL* aggressive non-Hodgkin lymphoma, *HL* Hodgkin lymphoma, *AML* acute myeloid leukemia, *ALL* acute lymphoblastic leukemia

^a^Allogeneic transplantation for MM, 9; iNHL/CLL, 24; MPN/CML, 219; MDS, 40; aNHL/HL, 23; AML/ALL, 239

^b^Time from last treatment in 1279 treated patients (82.5% of total patients)

^c^Follow-up provided by non-oncological internists (99 patients), general practitioners (94 patients), or others (10 patients) (Baum et al. 2023)

Based on the information provided, the patients were allocated to four types of follow-up care: university hospital oncologists, external oncologists, non-oncological internists or general practitioners, and no follow-up care (Table 1).

### General health care

1447 of 1484 responding survivors (97.5%) indicated to have a general practitioner who had been identical before and after blood cancer in 790 of 1388 survivors (56.9%) with a general practitioner at both times. General health care including disease prevention was provided exclusively by a general practitioner for 1,100 of 1,504 responding survivors (73.1%), by a blood cancer follow-up oncologist from the university hospital for 101 survivors (6.7%), by an external follow-up oncologist for 24 survivors (1.6%), by a university follow-up oncologist together with a general practitioner for 121 survivors (8.1%), by an external follow-up oncologist together with a general practitioner for 35 survivors (2.3%), by another specialist (most often a family member) for 12 survivors (0.8%), and by a general practitioner together with another specialist for 67 survivors (4.5%), most often a gynecologist (15 survivors), cardiologist (11 survivors), dermatologist (7 survivors), or urologist (7 survivors). Forty-four survivors (2.9%) reported not to undergo general health care.

Utilization of general health care was similar among survivors whose hematologic malignancy was followed up by a university hospital oncologist (988 of 1014 responding survivors, 97.4%), by an external oncologist (220/228, 96.5%), or by a non-oncological internist or general practitioner (191/195, 97.9%). It was considerably lower in patients not undergoing follow-up care (61/67, 91.0%; p = 0.0200).

By pooling the data from university hospital oncologists and external oncologists, we created three groups with different preventive care providers: general practitioners alone for 1100 survivors, oncologists alone for 125, and general practitioners together with oncologists for 156. These groups were compared in the subsequent analyses (Tables 2, 3, 4). A presentation without pooling of different types of oncologists is provided in the Online Supplementary Material (Supplementary Tables 1, 2, 3).

**Table 2:** Cancer screening after blood cancer in relation to blood cancer treatment and preventive care provider

	Number of patients screened^a^/number of patients responding (%)			
Screening in relation to treatment	All groups	No AlloTx	AlloTx	p^b^
Digital rectal examination, all patients	747/1308 (57.1%)	488/840 (58.1%)	259/468 (55.3%)	0.3348
Fecal occult blood test, all patients	775/1353 (57.2%)	492/861 (57.1%)	283/492 (57.5%)	0.8926
Colonoscopy, all patients	690/1375 (50.2%)	457/884 (51.7%)	233/491 (47.5%)	0.1317
Skin cancer screening, all patients	981/1400 (70.1%)	572/890 (64.3%)	409/510 (80.2%)	< 0.0001
Clinical breast examination, female patients	616/673 (91.5%)	385/425 (90.6%)	231/248 (93.1%)	0.2504
Mammography, female patients	483/663 (72.9%)	296/414 (71.5%)	187/249 (75.1%)	0.3124
Cervical smear, female patients	539/636 (84.7%)	331/395 (83.8%)	208/241 (86.3%)	0.3931
PSA test, male patients	387/728 (53.2%)	266/478 (55.6%)	121/250 (48.4%)	0.0627

Screening in relation to care provider	GP alone	Oncologist alone	GP and oncologist	p^c^
Digital rectal examination, all patients	570/952 (59.9%)	51/105 (48.6%)	69/133 (51.9%)	0.0267
Male patients	333/522 (63.8%)	28/60 (46.7%)	39/69 (56.5%)	0.0241
Female patients	237/430 (55.1%)	23/45 (51.1%)	30/64 (46.9%)	0.4347
Fecal occult blood test, all patients	578/971 (59.5%)	58/113 (51.3%)	72/144 (50.0%)	0.0350
Male patients	315/543 (58.0%)	34/68 (50.0%)	33/74 (44.6%)	0.0559
Female patients	263/428 (61.4%)	24/45 (53.3%)	39/70 (55.7%)	0.4182
Colonoscopy, all patients	509/991 (51.4%)	47/113 (41.6%)	68/145 (46.9%)	0.1060
Male patients	279/546 (51.1%)	30/67 (44.8%)	34/74 (45.9%)	0.4771
Female patients	230/445 (51.7)	17/46 (37.0%)	34/71 (47.9%)	0.1524
Skin cancer screening, all patients	725/1012 (71.6%)	72/113 (63.7%)	106/148 (71.6%)	0.2087
Male patients	399/559 (71.4%)	38/68 (55.9%)	52/75 (69.3%)	0.0319
Female patients	326/453 (72.0%)	34/45 (75.6%)	54/73 (74.0%)	0.8372
Clinical breast examination, female patients	444/480 (92.5%)	39/48 (81.3%)	68/75 (90.7%)	0.0028
Mammography, female patients	357/477 (74.8%)	25/46 (54.3%)	55/75 (73.0%)	0.0113
Cervical smear, female patients	386/455 (84.8%)	34/45 (75.6%)	64/72 (88.9%)	0.1448
PSA test, male patients	302/530 (58.0%)	26/64 (40.6%)	33/71 (46.5%)	0.0174

*AlloTx* allogeneic transplantation, *GP* general practitioner, *PSA* prostate-specific antigen, *p* chi^2^ test

^a^Number of patients undergoing the procedure (repeated and one-time-only examinations combined)

^b^No AlloTx versus AlloTx

^c^GP alone versus oncologist alone versus GP and oncologist

**Table 3:** Cardiovascular screening after blood cancer in relation to blood cancer treatment and preventive care provider

	Number of patients screened^a^/number of patients responding (%)			
Screening in relation to treatment	All groups	No AlloTx	AlloTx	p^b^
Blood pressure measurement^c^	597/835 (71.5%)	356/536 (66.4%)	241/299 (80.6%)	< 0.0001
Urine glucose measurement^c^	635/1195 (53.1%)	411/778 (52.8%)	224/417 (53.7%)	0.7690
Blood lipid measurement	1111/1504 (73.9%)	678/965 (70.3%)	433/539 (80.3%)	< 0.0001
Information about overweight	1016/1493 (68.1%)	657/960 (68.4%)	359/533 (67.4%)	0.6672
Weight reduction measures^d^	81/774 (10.5%)	57/534 (10.7%)	24/240 (10.0%)	0.7769
Advice to stop smoking^d^	225/329 (68.4%)	156/219 (71.2%)	69/110 (62.7%)	0.1175

Screening in relation to care provider	GP alone	Oncologist alone	GP and oncologist	p^b^
Blood pressure measurement^c^	408/594 (68.7%)	54/78 (69.2%)	56/83 (67.5%)	0.9677
Urine glucose measurement^c^	440/828 (53.1%)	43/93 (46.2%)	60/113 (53.1%)	0.4459
Blood lipid measurement	800/1082 (73.9%)	88 /121 (72.7%)	126/153 (82.4%)	0.0696
Information about overweight	739/1077 (68.6%)	80/121 (66.1%)	111/152 (73.0%)	0.4305
Weight reduction measures^d^	63/579 (10.9%)	5/51 (9.8%)	8/81 (9.9%)	0.9417
Advice to stop smoking^d^	167/239 (69.9%)	16/24 (66.7%)	28/36 (77.8%)	0.5677

*AlloTx* allogeneic transplantation, *GP* general practitioner, *p* chi^2^ test

^a^Number of patients undergoing the procedure (repeated and one-time-only events combined)

^b^No AlloTx versus AlloTx

^c^Number of patients screened/number of patients responding and not diagnosed with hypertension or diabetes, respectively

^d^Number of patients screened/number of patients responding and being overweight or smoker, respectively

^e^GP alone versus oncologist alone versus GP and oncologist

**Table 4:** Vaccination after blood cancer in relation to blood cancer treatment and preventive care provider

	Number of patients vaccinated/number of patients responding^a^ (%)			
Vaccination in relation to treatment	All groups	No Allo Tx	AlloTx	p^b^
Verification of vaccination status	673/1516 (44.4%)	206/975 (21.1%)	467/541 (86.3%)	< 0.0001
Influenza (yearly)	758/1437 (52.7%)	402/923 (43.6%)	356/514 (69.3%)	< 0.0001
*Streptococcus pneumoniae*	525/1509 (34.8%)	193/974 (19.8%)	332/535 (62.1%)	< 0.0001
Diphtheria/tetanus (every 10 years)	927/1508 (61.5%)	516/974 (52.9%)	411/ 534 (77.0%)	< 0.0001

Vaccination in relation to care provider	GP alone	Oncologist alone	GP and oncologist	p^b^
Verification of vaccination status	456/1087 (42.0%)	68/125 (54.4%)	91/155 (58.7%)	< 0.0001
Influenza (yearly)	530/1024 (51.8%)	63/120 (52.5%)	82/151 (54.3%)	0.8396
*Streptococcus pneumoniae*	356/1084 (32.8%)	47/123 (38.2%)	82/153 (53.6%)	< 0.0001
Diphtheria/tetanus (every 10 years)	666/1086 (61.3%)	73/124 (58.9%)	111/152 (73.0%)	0.0142

*AlloTx* allogeneic transplantation, *GP* general practitioner, *p* chi^2^ test

^a^Exclusion of patients with contraindication to vaccination

^b^No AlloTx versus AlloTx

^c^GP alone versus oncologist alone versus GP and oncologist

### Cancer screening

The survivors were asked whether they had been screened for other cancers after the diagnosis of blood cancer. The most frequent screening intervention was clinical breast examination (616 of 673 responding survivors, 91.5%), and the least frequent was colonoscopy (690/1375, 50.2%) (Table 2). There were no statistically significant differences between survivors with or without a history of allogeneic transplantation, except for skin cancer screening that predominated in transplanted survivors (Table 2). For most procedures, the screening rates were highest when preventive care was exclusively delivered by a general practitioner, and lowest when it was delivered by an oncologist, with intermediate rates for care delivered by both disciplines (Table 2). Statistical significance was reached for digital rectal examination, fecal occult blood testing, clinical breast examination, mammography, and PSA testing. For procedures performed in both sexes, differences between general practitioners and oncologists tended to be more pronounced in men than in women.

Table 5 details the survivors’ participation in statutory cancer screening programs in relation to the timepoint of becoming eligible for a program (before or after the blood cancer diagnosis). The participation rates ranged from 61.9% (digital rectal examination) to 92.1% (clinical breast examination). Breast cancer screening rates were significantly higher in women who became eligible for the program after (as compared to before) blood cancer. The converse was true for colorectal cancer screening rates in men (Table 5).

**Table 5:** Utilization of statutory cancer screening, cardiovascular screening and vaccination programs in eligible blood cancer survivors in relation to the timepoint of becoming eligible for the program

Statutory program (eligibility age limit)	Number of patients affected^a^/number of patients responding (%)			p^b^
	All patients	Timepoint of becoming eligible for the program		
		Before blood cancer	After blood cancer	
Cancer screening				
Skin cancer (≥ 35 years)	916/1289 (71.1%)	714/1008 (70.8%)	202/281 (71.9%)	0.7307
Fecal occult blood (≥ 50 years)	651 /925 (70.4%)	356/515 (69.1%)	295/410 (72.0%)	0.3499
Colonoscopy, male (≥ 50 years)	555/859 (64.6%)	208/309 (67.3%)	115/209 (55.0%)	0.0046
Colonoscopy, female (≥ 55 years)		114/166 (68.7%)	118/175 (67.4%)	0.8052
Digital rectal examination, male (≥ 45 years)	359/580 (61.9%)	213/345 (61.7%)	146/235 (62.1%)	0.9246
Cervical smear, female (≥ 20 years)	539/627 (86.0%)	517/600 (86.2%)	22/27 (81.5%)	0.4930
Clinical breast examination, female (≥ 30 years)	593/644 (92.1%)	222/254 (87.4%)	371/390 (95.1%)	< 0.0001
Mammography, female (≥ 50 years)	400/461 (86.8%)	209/251 (83.3%)	191/210 (91.0%)	0.0153
Cardiovascular screening (≥ 35 years)				
Blood pressure measurement^c^	516/744 (69.4%)	402/557 (72.2%)	114/187 (61.0%)	0.0040
Urine glucose measurement^c^	578/1062 (54.4%)	491/843 (58.2%)	87/219 (39.7%)	< 0.0001
Blood lipid measurement	1069/1393 (76.7%)	870/1108 (78.5%)	199/285 (69.8%)	0.0019
Information about overweight	947/1334 (71.0%)	749/1097 (68.3%)	198/237 (83.5%)	< 0.0001
Weight reduction measures^d^	78/740 (10.5%)	56/592 (9.5%)	22/148 (14.9%)	0.0554
Advice to stop smoking^d^	203/298 (68.1%)	159/231 (68.8%)	44/67 (65.7%)	0.6251
Vaccination (≥ 60 years)				
Influenza	381/669 (57.0%)	172/276 (62.3%)	209/393 (53.2%)	0.0118
* Streptococcus pneumoniae*	243/657 (37.0%)	103/282 (36.5%)	140/375 (37.3%)	0.8317

*p* chi^2^ test

^a^Number of patients undergoing the procedure (repeated and one-time-only events combined)

^b^Comparison of patients becoming eligible before or after the blood cancer diagnosis

^c^Number of patients screened/number of patients responding and not diagnosed with hypertension or diabetes, respectively

^d^Number of patients screened/number of patients responding and being overweight or smoker, respectively

Nine hundred and twenty seven of 1477 responding survivors (62.8%) reported not to be reminded of cancer screening. 247 of 1072 survivors (23.0%) cared for by a general practitioner were reminded by the general practitioner, 36 of 120 survivors (30.0%) treated by an oncologist were reminded by the oncologist, and 68 of 153 survivors (44.4%) treated by both a general practitioner and an oncologist were reminded by either the general practitioner, the oncologist, or both (p < 0.0001).

### Cardiovascular screening

Six hundred and ten of 1503 responding survivors (40.6%) reported to suffer from arterial hypertension and 198 of 1485 (13.3%) from diabetes. 719 of 1493 responding survivors (48.1%) stated not to be overweight, and 1177 of 1506 (78.2%) to be non-smokers. Allogeneic transplant recipients significantly more often denied overweight than did non-transplant patients (291/554 [52.5%] versus 428/997 [42.9%]; p = 0.0003). Other risk factors did not significantly differ between transplanted and non-transplanted survivors (data not shown). There were no statistically significant differences in the prevalence of hypertension, diabetes, overweight, or smoking between survivors receiving preventive care by general practitioners versus oncologists (data not shown).

Screening rates were highest for blood lipids (1111 of 1504 survivors, 73.9%) and lowest for urine glucose (635 of 1195 non-diabetic survivors, 53.1%) (Table 3). Weight reduction was initiated in 81 of 774 overweight survivors (10.5%), and an advice to stop smoking was given to 225 of 329 smokers (68.4%). Hypertension and hyperlipidemia were more often investigated in allogeneic transplant recipients than in non-transplanted patients. There were no statistically significant differences between survivors receiving preventive care by general practitioners alone, oncologists alone, or general practitioners and oncologists (Table 3).

Table 5 details the screening rates for selected interventions included in the statutory cardiovascular screening program (age limit, 35 years). Blood pressure, urinary glucose and blood lipid tests were significantly more often performed in survivors who had already been eligible for the program before the blood cancer diagnosis, while information about the risks of overweight was more often provided to survivors who became eligible after the diagnosis.

### Vaccination

The overall vaccination status was systematically verified in 673 of 1516 responding survivors (44.4%) and in 673 of 1352 responding survivors (49.8%) treated by (immuno) chemotherapy with or without stem cell transplantation. Vaccinations against influenza (yearly), *Streptococcus pneumoniae* (at least once), and diphtheria and tetanus (every 10 years) were performed in 758 of 1437 (52.7%), 525 of 1509 (34.8%), and 927 of 1508 responding survivors (61.5%), respectively (Table 4). Survivors with a history of allogeneic transplantation reported significantly higher vaccination rates than non-transplanted survivors. Except for diphtheria and tetanus, vaccination rates were lowest among survivors receiving preventive care by a general practitioner alone, and highest among survivors treated by both a general practitioner and an oncologist (Table 4).

Table 5 details the vaccination rates for influenza and *Streptococcus pneumoniae* in the respective statutory programs (age limit, 60 years). Survivors becoming eligible for statutory vaccination after blood cancer were significantly less likely to be vaccinated against influenza than survivors who had already been eligible before.

## Discussion

The major results of the preventive care part of the ABC study are the following: First, although blood cancer follow-up care was provided by an oncologist in more than 80% of survivors, almost all had a general practitioner in addition. Second, in almost three quarters of survivors, preventive care was exclusively delivered by a general practitioner. In about 10%, it was delivered by an oncologist, and in a similar percentage by a general practitioner together with an oncologist. Third, there were significant differences in cancer screening and vaccination rates between survivors treated by different disciplines. And fourth, participation in statutory disease prevention programs was high.

The preventive care interventions analyzed can be grouped into three categories: Measures that can be performed by any physician at any time, such as taking the blood pressure, ordering laboratory tests, or advising the patient to stop smoking; measures that require more time and experience, such as clinical breast examination, digital rectal examination, overweight counseling, or vaccination; and measures for which the patient has to be referred to a specialist, such as colonoscopy, mammography, cervical smear, or skin cancer screening. Our questionnaire allowed us to identify the physicians responsible for preventive care, but it did not specify to what extent the interventions were performed by the care providers themselves. In many instances, they were likely to play an organizational role, ensuring that the patient received the recommended care.

While cardiovascular screening rates were similar in survivors receiving preventive care by different providers, cancer screening was more often performed by general practitioners than by oncologists. This is consistent with a report from the USA where cancer survivors expected their follow-up physicians to provide preventive care irrespective of their medical qualification. While most general practitioners agreed to this view, only a minority of oncologists considered cancer screening their responsibility (Cheung et al. 2009). In contrast to cancer screening, vaccination rates were lowest when preventive care was exclusively delivered by a general practitioner. Physicians without oncological training may not be aware of the importance of vaccination for hematological patients. Improvement of communication between cancer physicians and primary care providers has repeatedly been identified as an area of unmet needs for cancer survivors (National Research Council 2006; Lagergren et al. 2019; Gallicchio et al. 2021). Our study adds to the evidence that this is true not only for solid tumors, but also for blood cancer (Forsythe et al. 2014).

Survivors not undergoing blood cancer follow-up care significantly less often received preventive care than survivors on follow-up care. Thus, the decision to abstain from medical care appeared to extend to several areas of health care. Neglect of more than one area has previously been observed in individuals abstaining from breast cancer screening (Mayer-Oakes et al. 1996).

Allogeneic transplant recipients are at particularly high risk for a variety of late effects (Bishop et al. 2010; Saunders et al. 2020; Armenian et al. 2022). Immunosuppression increases the risk of infection, conditioning by total-body irradiation and immunosuppression increase the risk of skin cancer, and immunosuppressive drugs may raise blood pressure, glucose and lipid levels (Schwartz et al. 2009; Bhatia et al. 2017). These facts explain the spectrum of preventive procedures that were more consistently performed in transplanted as compared to non-transplanted survivors. The most pronounced differences were found for vaccination rates.

Starting at a predefined age limit, the German health care system provides statutory programs for cancer screening, cardiovascular screening, and vaccination. Utilization of these programs in the general population was investigated in the questionnaire-based part of the ‘German Adult Health Study 1’ (‘Studie zur Gesundheit Erwachsener in Deutschland’, DEGS1) that was performed from 2008 to 2011 (Gößwald et al. 2013). Utilization of skin cancer screening was 24.4% (ABC study, 71.1%), fecal occult blood testing 53.0% (ABC study, 70.4%), colonoscopy 54.8% (ABC study, 64.6%), digital rectal examination in males 38.9% (ABC study, 61.9%), cervical smear 52.8% (ABC study, 86.0%), clinical breast examination 62.1% (ABC study, 92.1%), and mammography 71.3% (ABC study, 86.8%) (Starker and Saß 2013). These rates are considerably lower than those reported in the ABC study. The discrepancy may in part be explained by differences in the periods analyzed—depending on the procedure, cancer screening within the last year, the last two years, or the last ten years in DEGS1 as opposed to any time after blood cancer diagnosis in the ABC study. The extent of the observed differences, however, suggests that utilization of cancer screening by blood cancer survivors was at least as high as in the general population.

Our observations are at variance with registry-based reports from Canada, where colorectal cancer screening was recorded in only 23–38%, cervical cancer screening in 68–80%, and breast cancer screening in 56–68% of Hodgkin lymphoma survivors (Hodgson et al. 2010; Grunfeld et al. 2012). Our results resemble questionnaire-based studies from the USA, where Hodgkin lymphoma and hematopoietic transplant survivors had cancer screening rates that were similar to non-cancer controls (Ng et al. 2008; Bishop et al. 2010), and non-Hodgkin-lymphoma survivors had higher rates than controls (Pophali et a. 2020). Meta-analyses of studies in solid tumors also came to the conclusion that cancer screening is significantly more often performed in cancer survivors than in individuals without a history of cancer (Corkum et al. 2013; Uhlig et al. 2018). Disparities between studies are likely to be related to differences in socio-economic factors, health care systems, and methods.

In the years immediately preceding the ABC study, the statutory cardiovascular screening program ‘Gesundheits-Check-up’ (‘health check-up’) was utilized by 35.1–42.0% of eligible insured individuals in Germany (Statista Research Department 2022). Details of individual tests are not available. With regard to the most common tests (blood pressure, urine glucose, blood lipids, information about overweight), blood cancer survivors from the ABC study underwent screening procedures considerably more often (69.4%, 54.4%, 76.7%, and 71.0%, respectively) than the general population did. Again, the periods analyzed differed. While the general population data pertain to a single year, patients in the ABC study were asked whether these tests were performed ‘regularly’.

Published data on cardiovascular screening in blood cancer survivors is scant. Our results are in line with a report from the USA where the blood pressure was significantly more often measured in survivors with a history of allogeneic transplantation than in non-cancer controls (96.1% versus 90.2%) (Bishop et al. 2010). They differ from a Canadian report where ‘periodic health examinations’ were less often performed in Hodgkin lymphoma survivors than in controls (56.4% versus 59.0%) (Grunfeld et al. 2012), and from reports in solid tumors where both blood pressure (Khan et al. 2010) and blood lipid tests (Earle et al. 2004; Lafata et al. 2015) were found to be underused in long-term survivors.

DEGS1 also investigated the utilization of statutory vaccination against *Streptococcus pneumoniae* and influenza (Poethko-Müller and Schmitz 2013). In the Western parts of Germany, the vaccination rates for *Streptococcus pneumoniae* were 24.6% for men and 27.2% for women, which is about 10 percentage points lower than in the ABC study (37.0%). For influenza, the respective figures in the general population (men, 64.5%; women, 65.3%) were somewhat higher than in the ABC study (57.0%). Comparisons need to be done with caution, because DEGS1 investigated the lifetime prevalence, while the ABC study asked about ‘yearly’ influenza vaccinations.

In an interview-based study from the USA, the influenza vaccination rate among blood cancer survivors was in the same range (men, 58.1%; women, 59.2%) as in the ABC study (Chang et al. 2021). In a questionnaire-based American study restricted to non-Hodgkin lymphoma survivors, it was considerably higher than in the general population (82% versus 52%) (Pophali et al. 2020). Data from France are heterogeneous. In a questionnaire-based study restricted to non-transplanted hematological patients from Poitiers, the vaccination rates for influenza (32.3%) and *Streptococcus pneumoniae* (11.2%) were low (Monier et al. 2020). By contrast, in a similar study from Besancon, the self-reported vaccination rates for influenza and *Streptococcus pneumoniae* were 52% and 45% (Pierron et al. 2021), which was higher than in ABC study participants without a history of transplantation (43.6% and 19.8%). The study from Besancon highlighted the pivotal role of the method used to determine the vaccination rate. While 45% of survivors claimed to have been vaccinated against *Streptococcus pneumoniae*, this was confirmed by medical documents in only 19%.

Limitations of the ABC-study are its reliance on patient reporting which is subject to participation and recall biases, and the exclusion of patients who died before the study was conducted (prevalence-incidence bias). Patients alive at the time of the survey are a selection of individuals with a favorable disease course. Whether the information provided by them is representative of the entire population, remains uncertain. A further limitation is the fact that any comparison of care providers may be confounded by differences in disease spectrum and follow-up duration. In addition, the term ‘general practitioner’ remained vaguely defined. In our previous analysis (Baum et al. 2023), patients often were uncertain about the medical background of their ‘general practitioner’. This was not surprising because general practitioners and internists have overlapping roles in the German health care system. Thus, the term ‘general practitioner’ is likely to include other disciplines, in particular non-oncological internists. A strength of the ABC study is its large size with a high proportion of patients in late periods of follow-up care.

## Conclusions

In most blood cancer survivors participating in the ABC study, preventive care was delivered by a general practitioner. Utilization rates of statutory disease prevention programs appeared similar to or higher than in the general population. Cancer screening rates were higher in survivors receiving preventive care by general practitioners than in survivors cared for by oncologists. The converse was true for vaccination rates. To ensure widespread delivery of preventive care for blood cancer survivors and avoid redundancy, communication between oncologists and primary care providers needs to be improved.

## Supplementary Information

Below is the link to the electronic supplementary material.

Supplementary file1 (PDF 241 KB)

## Data Availability

The datasets used and analyzed during this study are available from the corresponding author on reasonable request.
