# Supplementary material for: Preventive health care in blood cancer survivors: results from the ABC study
Source: J Cancer Res Clin Oncol. 2023 Jul 3;149(13):11531–40. doi: 10.1007/s00432-023-04984-9 (PMC10465397; doi:10.1007/s00432-023-04984-9)
Supplement: Supplementary file 1 — Supplementary file1 (PDF 241 KB) [file 432_2023_4984_MOESM1_ESM.pdf]

Supplementary material

Preventive health care in blood cancer survivors –

results from the ABC study

Julia Baum,[1] Hildegard Lax,[2] Nils Lehmann,[2] Anja Merkel-Jens,[2] Dietrich W. Beelen,[3]

Karl-Heinz Jöckel,[2] Ulrich Dührsen[1]

[1] Klinik für Hämatologie, Universitätsklinikum Essen, Universität Duisburg-Essen, Germany

[2] Institut für Medizinische Informatik, Biometrie und Epidemiologie, Universität Duisburg-Essen, Germany

[3] Klinik für Knochenmarktransplantation, Universitätsklinikum Essen, Universität Duisburg-Essen, Germany

Supporting information

Supplementary Table 1 (Cancer screening by preventive care provider)

Supplementary Table 2 (Cardiovascular screening by preventive care provider)

Supplementary Table 3 (Vaccination by preventive care provider)

Patient questionnaire (19 questions related to preventive care)

Supplementary Table 1: Cancer screening after blood cancer in relation to preventive care provider

Screening procedure	Number of patients screened[a] / number of patients responding (%)					p
	**General practitioner (GP) alone**	**University hospital oncologist alone**	**External oncologist alone**	**University hospital oncologist and GP**	**External oncologist and GP**	
Digital rectal examination – all patients	570 / 952 (59.9%)	43 / 84 (51.2%)	8 / 21 (38.1%)	52 / 101 (51.5%)	17 / 32 (53.1%)	0.0762
Male	333 / 522 (63.8%)	24 / 46 (52.3%)	4 / 14 (28.6%)	31 / 54 (57.4%)	8 / 15 (53.3%)	0.0395
Female	237 / 430 (55.1%)	19 / 38 (50.0%)	4 / 7 (57.1%)	21 / 47 (44.7%)	9 / 17 (52.9%)	0.7120
Fecal occult blood test – all patients	578 / 971 (59.5%)	49 / 91 (53.4%)	9 / 22 (40.9%)	55 / 111 (49.5%)	17 / 33 (51.5%)	0.0932
Male	315 / 543 (58.0%)	30 / 53 (56.6%)	4 /15 (26.7%)	27 / 58 (46.6%)	6 / 16 (37.5%)	0.0337
Female	263 / 428 (61.4%)	19 / 38 (50.0%)	5 / 7 (71.4%)	28 / 53 (52.8%)	11 / 17 (64.7%)	0.4582
Colonoscopy – all patients	509 / 991 (51.4%)	40 / 92 (43.5%)	7 / 21 (33.3)	47 / 110 (42.7%)	21 / 35 (60.0%)	0.0792
Male	279 / 546 (51.1%)	28 / 53 (52.8%)	2 / 14 (14.3%)	24 / 57 (42.1%)	10 / 17 (58.8%)	0.0492
Female	230 / 445 (51.7%)	12 / 39 (30.8%)	5 / 7 (71.4%)	23 / 53 (43.4%)	11 / 18 (61.1%)	0.0524
Skin cancer screening – all patients	725 / 1,012 (71.6%)	60 / 93 (64.5%)	12 / 20 (60.0%)	84 / 116 (72.4%)	22 / 32 (68.8%)	0.4840
Male	399 / 559 (71.4%)	31 / 54 (57.4%)	7 / 14 (50.0%)	43 / 60 (71.7%)	9 / 15 (60.0%)	0.0933
Female	326 / 453 (72.0%)	29 / 39 (74.4%)	5 / 6 (83.3%)	41 / 56 (73.2%)	13 / 17 (76.5%)	0.9591
Breast palpation – female patients	444 / 480 (92.5%)	32 / 40 (80.0%)	7 / 8 (87.5%)	51 / 57 (89.5%)	17 / 18 (94.4%)	0.0927
Mammography – female patients	357 / 477 (74.8%)	20 / 39 (51.3%)	5 / 7 (71.4%)	41 / 58 (70.7%)	14 / 17 (82.4%)	0.0255
Cervical smear – female patients	386 / 455 (84.8%)	28 / 37 (75.7%)	6 / 8 (75.0%)	48 / 55 (87.3%)	16 / 17 (94.1%)	0.3627
PSA test – male patients	302 / 530 (58.0%)	20 / 50 (40.0%)	6 / 14 (42.9%)	23 / 54 (42.6%)	10 / 17 (58.8%)	0.0494

PSA, prostate-specific antigen; p, chi^2 test

[a] Number of patients undergoing the procedure (repeated and one-time-only examinations combined)

Supplementary Table 2: Cardiovascular screening after blood cancer in relation to preventive care provider

Screening procedure	Number of patients screened[a] / number of patients responding (%)					p
	**General practitioner (GP) alone**	**University hospital oncologist alone**	**External oncologist alone**	**University hospital oncologist and GP**	**External oncologist and GP**	
Blood pressure measurement[b]	408 / 594 (68.7%)	42 / 59 (71.2%)	12 / 19 (63.2%)	44 / 60 (73.3%)	12 / 23 (52.2%)	0.4123
Urine glucose measurement[b]	440 / 828 (53.1%)	34 / 73 (46.6%)	9 / 20 (45.0%)	46 / 84 (54.8%)	14 / 29 (48.3%)	0.7368
Blood lipid measurement	800 / 1,082 (73.9%)	75 / 98 (76.5%)	13 / 23 (56.5%)	98 / 118 (83.1%)	28 / 35 (80.0%)	0.0515
Information about overweight	739 / 1,077 (68.6%)	64 / 97 (66.0%)	16 / 24 (66.7%)	86 / 117 (73.5%)	25 / 35 (71.4%)	0.7827
Weight reduction measures[c]	63 / 579 (10.9%)	4 / 44 (9.1%)	1 / 7 (14.3%)	7 / 60 (11.7%)	1 / 21 (4.8%)	0.8994
Advice to stop smoking[c]	167 / 239 (69.9%)	13 / 20 (65.0%)	3 / 4 (75.0%)	21 / 27 (77.8%)	7 / 9 (77.8%)	0.8626

p, chi^2 test

[a] Number of patients undergoing the procedure (repeated and one-time-only events combined)

[b] Number of patients screened / number of patients responding and not diagnosed with hypertension or diabetes, respectively

[c] Number of patients screened / number of patients responding and being overweight or smoker, respectively

Supplementary Table 5: Vaccination after blood cancer in relation to preventive care provider

Vaccination	Number of patients vaccinated / number of patients responding[a] (%)					p
	**General practitioner (GP) alone**	**University hospital oncologist alone**	**External oncologist alone**	**University hospital oncologist and GP**	**External oncologist and GP**	
Verification of vaccination status	456 / 1,087 (42.0%)	58 / 101 (57.4%)	10 / 24 (41.7%)	76 / 120 (63.3%)	15 / 35 (42.9%)	<0.0001
Influenza (yearly)	530 / 1,024 (51.8%)	54 / 96 (56.3%)	9 / 24 (37.5%)	74 / 118 (62.7%)	8 / 33 (24.2%)	0.0011
Streptococcus pneumoniae	356 / 1,084 (32.8%)	43 / 99 (43.4%)	4 / 24 (16.7%)	64 / 118 (54.2%)	18 / 35 (51.4%)	<0.0001
Diphtheria/tetanus (every 10 years)	666 / 1,086 (61.3%)	62 / 100 (62.0%)	11 / 24 (45.8%)	87 / 117 (74.4%)	24 / 35 (68.6%)	0.0260

p, chi^2 test

[a] Exclusion of patients with contraindication to vaccination

Aftercare in blood cancer survivors (ABC study)

Retrospective part – Patient questionnaire

Questions related to preventive health care

1. **Haben Sie eine Hausärztin / einen Hausarzt?**

☐1 Ja
☐2 Nein

2. **Hatten Sie vor Ihrer Blutkrebserkrankung eine Hausärztin / einen Hausarzt?**

☐1 Ja, aber einen anderen
☐2 Ja, denselben
☐3 Nein

3. **Wer behandelt Sie in Bezug auf die allgemeine Gesundheitsfürsorge?**

☐1 Niemand
☐2 Meine Hausärztin / mein Hausarzt
☐3 Meine nachsorgende Fachärztin / mein nachsorgender Facharzt (sofern die Nachsorge nicht vom Hausarzt durchgeführt wird)
☐4 Eine andere Person oder medizinische Einrichtung, und zwar (bitte angeben): ____

Früherkennungsuntersuchungen für Krebserkrankungen

In den Fragen 4 und 5 geht es um die Teilnahme an Krebsfrüherkennungs-untersuchungen.

Hiermit sind Untersuchungen gemeint, die der frühzeitigen Erkennung einer anderen Krebs-erkrankung als der bei Ihnen bereits bekannten Blutkrebserkrankung dienen.

4. **Wurden bei Ihnen nach Abschluss der Behandlung** (bei intensiv behandlungs-bedürftigen Erkrankungen) **/ nach erstmaliger Feststellung der Blutkrebserkrankung** (bei nicht behandlungsbedürftigen oder dauerhaft mit Tabletten behandlungsbedürftigen Erkrankungen) **folgende Vorsorgeuntersuchungen für andere Krebserkrankungen durchgeführt?**

Art der Früherkennungsuntersuchung	Nein	Ja, einmal	Ja, mehrfach	Weiß ich nicht
Stuhluntersuchung auf Blut (Hämoccult-Test; Früherkennung von Darmkrebs)	☐1	☐2	☐3	☐4
Darmspiegelung (Koloskopie; Früherkennung von Darmkrebs)	☐1	☐2	☐3	☐4
Untersuchung der Haut auf Muttermale (Früherkennung von Hautkrebs)	☐1	☐2	☐3	☐4
Tastuntersuchung der Brustdrüsen durch eine Ärztin / einen Arzt (Früherkennung von Brustkrebs)	☐1	☐2	☐3	☐4
Röntgenuntersuchung der Brustdrüsen (Mammographie; Früherkennung von Brustkrebs)	☐1	☐2	☐3	☐4
Abstrich des Gebärmutterhalses (Früherkennung von Gebärmutterhalskrebs)	☐1	☐2	☐3	☐4
Untersuchung des Darmausgangs mit dem Finger (Früherkennung von Enddarm- und Vorsteherdrüsenkrebs, Prostatakrebs)	☐1	☐2	☐3	☐4
Bestimmung des PSA-Werts im Blut (Früherkennung von Vorsteherdrüsenkrebs, Prostatakrebs)	☐1	☐2	☐3	☐4

5. **Wer erinnert Sie an die Krebsfrüherkennungsuntersuchungen?**

☐1 Niemand
☐2 Meine Hausärztin / mein Hausarzt
☐3 Meine nachsorgende Fachärztin / mein nachsorgender Facharzt (sofern die Nachsorge nicht vom Hausarzt durchgeführt wird)
☐4 Jemand anderes,
und zwar (bitte angeben): ____

Vorsorge von Herz-Kreislauferkrankungen

Die Fragen 6-15 befassen sich mit der allgemeinen Gesundheitsvorsorge zur Verhinderung von Herz-Kreislauferkrankungen (insbesondere Herzinfarkt und Schlaganfall).

Risikofaktoren für Herz-Kreislauferkrankungen sind u. a. Bluthochdruck (Hypertonus, Hypertonie), Zuckerkrankheit (Diabetes mellitus), hohe Blutfettwerte (insbesondere Cholesterin), Übergewicht und Rauchen. Wir möchten wissen, ob diese Risikofaktoren bei Ihnen bestehen und ob Sie auf mögliche Folgeerkrankungen untersucht werden bzw. wurden.

6. **Ist bei Ihnen eine Zuckerkrankheit** (Diabetes mellitus) **bekannt?**

☐1 Ja,
und zwar seit (bitte angeben): ____
☐2 Nein
☐3 Weiß ich nicht

7. **Falls bei Ihnen keine Zuckerkrankheit bekannt ist, werden Blut- oder Urinuntersuchungen durchgeführt, um eine bisher nicht bekannte Zuckerkrankheit frühzeitig zu erkennen?**

☐1 Ja
☐2 Nein
☐3 Weiß ich nicht
☐4 Die Frage trifft auf mich nicht zu, da bei mir eine Zuckerkrankheit bereits bekannt ist

8. **Eine Zuckerkrankheit kann neben Herz-Kreislauferkrankungen auch Schäden an den Nieren, Augen, Nerven und Füßen nach sich ziehen. Welche Untersuchungen werden zur Früherkennung dieser Folgeerkrankungen durchgeführt?**
(Hier sind mehrere Antworten möglich!)

☐1 Keine
☐2 Urinuntersuchung
☐3 Kontrolle der Nierenwerte im Blut
☐4 Untersuchung durch eine Augenärztin / einen Augenarzt
☐5 Untersuchung durch eine Nervenärztin / einen Nervenarzt (Neurologe)
☐6 Untersuchung der Füße (anschauen, abtasten)
☐7 Sonstiges,
und zwar (bitte angeben): ____
☐8 Die Frage trifft auf mich nicht zu, da bei mir keine Zuckerkrankheit vorliegt

9. **Ist bei Ihnen ein Bluthochdruck** (Hypertonus, Hypertonie) **bekannt?**

☐1 Ja,
und zwar seit (bitte angeben): ____
☐2 Nein
☐3 Weiß ich nicht

10. **Ein Bluthochdruck kann Schäden am Herzen, an den Blutgefäßen, Nieren und Augen nach sich ziehen. Welche Untersuchungen werden zur Früherkennung von Folgeschäden eines Bluthochdrucks durchgeführt?**
(Hier sind mehrere Antworten möglich!)

☐1 Elektrokardiogramm (EKG)
☐2 Belastungs-EKG
☐3 Echokardiographie (Herzultraschall, Herzecho)
☐4 Ultraschall (Doppler) der Halsgefäße
☐5 Urinuntersuchung
☐6 Kontrolle der Nierenwerte im Blut
☐7 Untersuchung durch eine Augenärztin / einen Augenarzt
☐8 Sonstiges,
und zwar (bitte angeben): ____
☐9 Die Frage trifft auf mich nicht zu, da ich keinen Bluthochdruck habe

11. **Falls kein Bluthochdruck bekannt ist, wird der Blutdruck regelmäßig gemessen, um einen bisher unbekannten Bluthochdruck frühzeitig zu erkennen?**

☐1 Ja
☐2 Nein
☐3 Weiß ich nicht
☐4 Die Frage trifft auf mich nicht zu, da bei mir ein Bluthochdruck bereits bekannt ist

12. **Werden Ihre Blutfettwerte** (z. B. Cholesterin) **regelmäßig bestimmt?**

☐1 Ja
☐2 Nein
☐3 Weiß ich nicht

13. **Wurden Sie von Ihrer Ärztin / Ihrem Arzt darauf hingewiesen, dass Übergewicht ein Risikofaktor für Herz-Kreislauferkrankungen ist?**

☐1 Ja
☐2 Nein
☐3 Weiß ich nicht

14. **Hat Ihre Ärztin / Ihr Arzt bei Ihnen Maßnahmen zum Abnehmen** (Gewichtsreduktion) **eingeleitet?**

☐1 Ja
☐2 Nein
☐3 Weiß ich nicht
☐4 Die Frage trifft auf mich nicht zu, da ich nicht übergewichtig bin

15. **Hat Ihre Ärztin / Ihr Arzt Sie aufgefordert, zur Vermeidung von Herz-Kreislauf- und Krebserkrankungen mit dem Rauchen aufzuhören?**

☐1 Ja
☐2 Nein
☐3 Weiß ich nicht
☐4 Die Frage trifft auf mich nicht zu, da ich Nichtraucher bin

Impfungen

Zur allgemeinen Gesundheitsfürsorge gehören auch Impfungen. Impfungen gegen Diphtherie und Tetanus werden für alle Altersklassen, Impfungen gegen Grippeviren und Pneumokokken (*Erreger schwerer Lungenentzündungen)* ***für einige besonders gefährdete Bevölkerungsgruppen empfohlen.***

Mit den Fragen 16-19 möchten wir herausfinden, ob die genannten Impfungen bei Ihnen durchgeführt werden bzw. wurden.

16. **Lassen Sie sich jährlich gegen Grippe impfen?**

☐1 Ja
☐2 Nein
☐3 Weiß ich nicht
☐4 Ich lasse mich nicht mehr gegen Grippe impfen, da ich frühere Grippeimpfungen schlecht vertragen habe
☐5 Ich darf mich nicht gegen Grippe impfen lassen, da ich eine Hühnereiweißallergie habe

17. **Wurde bei Ihnen eine Impfung gegen Pneumokokken** (Erreger schwerer Lungen-entzündungen) **durchgeführt?**

☐1 Ja
☐2 Nein
☐3 Weiß ich nicht

18. **Werden die bereits im Kindesalter vorgeschriebenen Impfungen gegen Diphtherie und Tetanus bei Ihnen etwa alle 10 Jahre aufgefrischt?**

☐1 Ja
☐2 Nein
☐3 Weiß ich nicht
☐4 Ich darf nicht gegen Diphtherie und Tetanus geimpft werden, da ich frühere Diphtherie- / Tetanus-Impfungen schlecht vertragen habe

19. **Wurde Ihr Impfschutz nach Abschluss der Chemotherapie, Antikörperbehandlung, Hochdosistherapie oder Knochenmarktransplantation überprüft?**

☐1 Ja
☐2 Nein
☐3 Weiß ich nicht
☐4 Die Frage trifft auf mich nicht zu, da ich keine Chemotherapie, Antikörperbehandlung, Hochdosistherapie oder Knochenmarktransplantation erhalten habe
